# How Biomaterials Can Influence Various Cell Types in the Repair and Regeneration of the Heart after Myocardial Infarction

**DOI:** 10.3389/fbioe.2016.00062

**Published:** 2016-07-18

**Authors:** Zachary Lister, Katey J. Rayner, Erik J. Suuronen

**Affiliations:** ^1^Division of Cardiac Surgery, University of Ottawa Heart Institute, Ottawa, ON, Canada; ^2^Department of Cellular and Molecular Medicine, University of Ottawa, Ottawa, ON, Canada; ^3^Atherosclerosis, Genomics and Cell Biology Group, University of Ottawa Heart Institute, Ottawa, ON, Canada

**Keywords:** biomaterials, myocardial infarction, cell response, cardiac regeneration, fibroblasts, macrophages, endothelial cells, cardiomyocytes

## Abstract

The healthy heart comprises many different cell types that work together to preserve optimal function. However, in a diseased heart the function of one or more cell types is compromised which can lead to many adverse events, one of which is myocardial infarction (MI). Immediately after MI, the cardiac environment is characterized by excessive cardiomyocyte death and inflammatory signals leading to the recruitment of macrophages to clear the debris. Proliferating fibroblasts then invade, and a collagenous scar is formed to prevent rupture. Better functional restoration of the heart is not achieved due to the limited regenerative capacity of cardiac tissue. To address this, biomaterial therapy is being investigated as an approach to improve regeneration in the infarcted heart, as they can possess the potential to control cell function in the infarct environment and limit the adverse compensatory changes that occur post-MI. Over the past decade, there has been considerable research into the development of biomaterials for cardiac regeneration post-MI; and various effects have been observed on different cell types depending on the biomaterial that is applied. Biomaterial treatment has been shown to enhance survival, improve function, promote proliferation, and guide the mobilization and recruitment of different cells in the post-MI heart. This review will provide a summary on the biomaterials developed to enhance cardiac regeneration and remodeling post-MI with a focus on how they control macrophages, cardiomyocytes, fibroblasts, and endothelial cells. A better understanding of how a biomaterial interacts with the different cell types in the heart may lead to the development of a more optimized biomaterial therapy for cardiac regeneration.

## Introduction

### Myocardial Infarction

**Figure 1:**
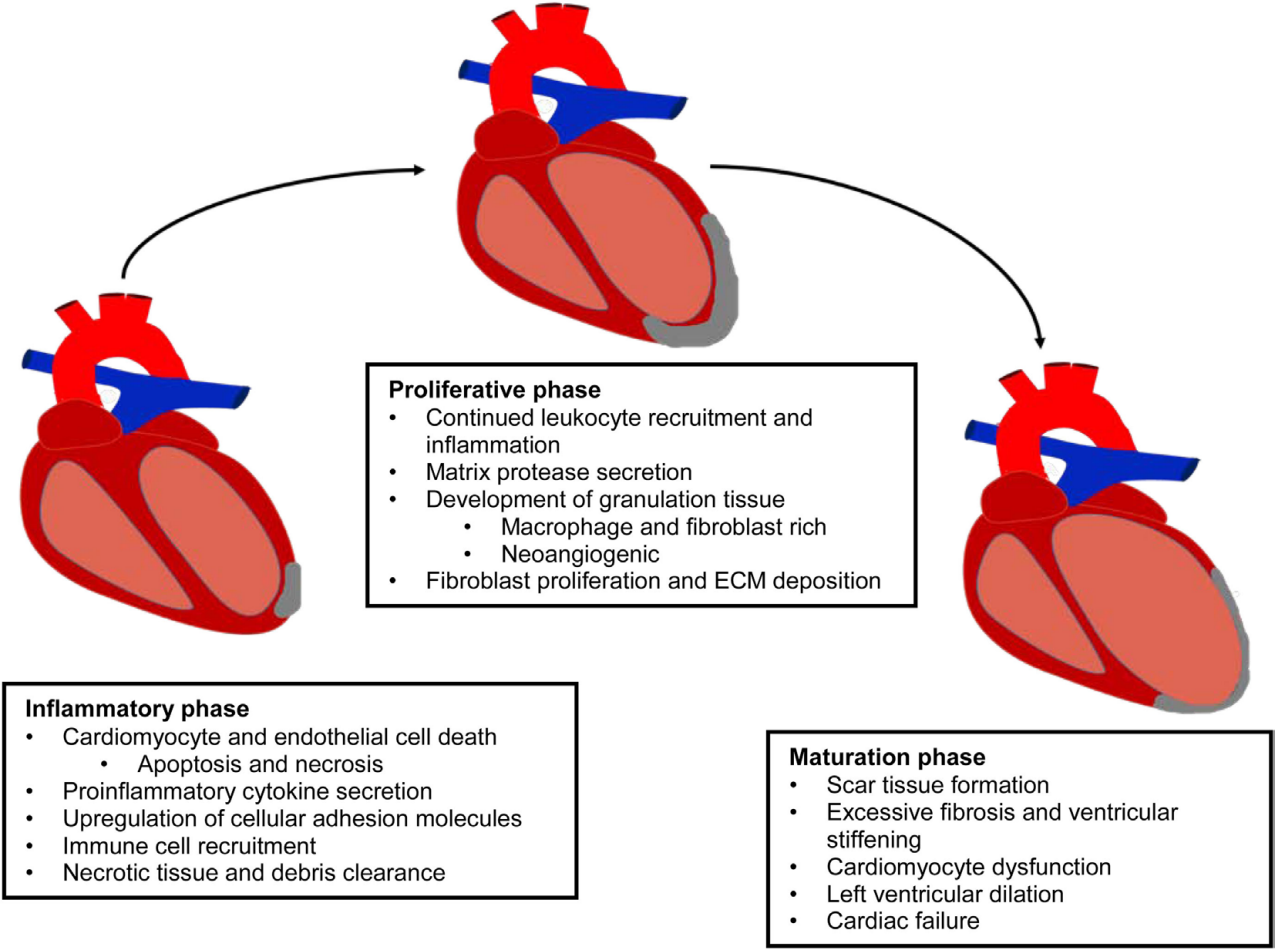
**Evolution of the infarcted myocardium**. MI results in the activation of a multiphase healing process, involving three overlapping phases: the inflammatory, proliferation, and maturation phases. This repair process prevents ventricular rupture, but it is insufficient to protect the myocardium from massive tissue loss and adverse remodeling.

Cardiovascular disease (CVD) is defined as a physiological condition that causes impaired function of the heart or blood vessels of the circulatory system. Myocardial infarction (MI) is often the consequence of prolonged CVD and results from the blockage of one or more coronary arteries causing ischemia (lack of oxygen and nutrients due to loss of blood supply) to the myocardium (Frangogiannis, 2008; Rane and Christman, 2011). The ischemic event triggers a switch from aerobic to anaerobic respiration leading to acidosis from lactic acid accumulation, the generation of reactive oxygen species (ROS), and a significant loss of cardiac myocytes (Dzau et al., 2006; Jourdan-Lesaux et al., 2010). The post-MI heart undergoes a complex multiphase healing process, involving three overlapping phases: the inflammatory, proliferation, and maturation phases (Pfeffer and Braunwald, 1990; Frangogiannis, 2006, 2008, 2012b; Fraccarollo et al., 2012) (Figure 1). In the inflammatory phase, apoptotic and necrotic cardiomyocytes release cytokines that signal for the recruitment of neutrophils and monocytes (Fraccarollo et al., 2012). Upon arrival of these immune cells to the ischemic tissue, there is a cascade of neurohormonal and intracellular signaling events that further mobilizes and activates more immune cells, namely neutrophils and macrophages, to remove debris from the infarct area (Frangogiannis, 2008; Fraccarollo et al., 2012; Sutton and Sharpe, 2015). After the removal of cellular debris, macrophages release growth factors and cytokines leading to the maturation of granulation tissue through angiogenesis and the promotion of fibroblast proliferation, marking the beginning of the transition from the inflammatory phase to the proliferation phase (Pfeffer and Braunwald, 1990; Fraccarollo et al., 2012). During the proliferative phase, fibroblasts migrate into the infarct where they respond to TGF-β1, fibronectin extra domain A, and mechanical tension causing them to undergo a phenotypic change to myofibroblasts (van den Borne et al., 2010; Dobaczewski et al., 2011; Fraccarollo et al., 2012; Sutton and Sharpe, 2015). During the maturation phase, these “new” myofibroblasts, which have many smooth muscle-like qualities including the ability to contract, secrete large amounts of extracellular matrix (ECM) proteins, primarily collagen, into the infarct. The deposition of ECM forms the collagenous scar, which serves as mechanical support to protect the heart from ventricular rupture and is termed reparative fibrosis (Fraccarollo et al., 2012; Sutton and Sharpe, 2015). Although this scar is necessary to prevent rupture, maturation of the scar introduces its own pathophysiologic complications. Initially, there is apoptosis of fibroblasts and vascular cells (Frangogiannis, 2008). The rigid scar, which does not contribute to the synchronous beating of the heart, leads to cardiac dilation, cardiac hypertrophy, and eventual cardiac failure (Pfeffer and Braunwald, 1990; Dzau et al., 2006; Jourdan-Lesaux et al., 2010; Fraccarollo et al., 2012; Kempf et al., 2012).

### Current Treatments for Myocardial Infarction

The primary goal in treating acute MI is to restore blood flow in an attempt to minimize the size of the infarct and decrease the amount of cellular trauma to cardiac tissue. Current medical practise generally consists of a combination of surgical or pharmacological techniques (Wijns et al., 2010; Steg et al., 2012). Primary surgical techniques utilized to reperfuse the system are the implantation of stents and coronary artery bypass surgery (Wijns et al., 2010). A variety of pharmacological agents are also used either to avoid or complement surgical techniques. Thrombolytics are an option to circumvent potentially invasive surgical methods if successful as they are designed to break up clots in order to restore blood flow (Antman et al., 2004). Other therapies include the use of vasodilators and blood thinners in order to promote blood flow and reduce the risk of further clot formation (Antman et al., 2004; Wijns et al., 2010; Steg et al., 2012). All these therapeutic techniques aim to restore cardiac perfusion while further medication is prescribed in order to manage patient symptoms and prevent further damage to the structural components of the heart. Despite the success of these treatments in saving and improving the quality of life of patients, they do not address the underlying cause of the disease and do not replace the tissue that is lost. Since the heart has a very low capacity for regeneration, new treatments are required in order to grow new tissue and heal the heart at the cellular and molecular level.

### Use of Biomaterials to Treat MI

Over the last decade, there are has been considerable research dedicated to the development of biomaterials, both synthetic and natural (see Table 1 for list of abbreviations), to aid in the healing process post-MI (Suuronen et al., 2006, 2009; Jourdan-Lesaux et al., 2010; Rane and Christman, 2011; Kuraitis et al., 2012; Ahmadi et al., 2014a). Biomaterials can take many forms from injectable hydrogels to solid patches and can serve a variety of purposes. A left ventricular restraint is a device that is surgically implanted on the outer wall of the heart in order to provide increased mechanical support to the weakened ventricle of the infarcted heart (Rane and Christman, 2011). Cardiac patches, which can be created in the lab and sutured or applied to the surface of the tissue at the site of myocardial injury, have also been employed. These integrate with the host tissue and can be used to deliver therapeutics such as drugs, growth factors, or small molecules (Rane and Christman, 2011). Recently, there has been an increasing amount of research into injectable hydrogels that can be delivered directly into the cardiac tissue. Although much research has been performed in the development of biomaterials to enhance cell transplantation therapies, it is increasingly evident that biomaterials can also be effective on their own without the cells, which is the focus of this review. Thus far, various biomaterials have been shown to improve left ventricular ejection fraction (LVEF), reduce infarct size and scarring, and enhance tissue viability, angiogenesis, and cardiomyogenesis. It is clear that biomaterials are capable of improving cardiac repair at a cellular level rather than just controlling the effects of deteriorating function (Venugopal et al., 2012; Jalil and Seliktar, 2015; Pascual-Gil et al., 2015; Wang and Christman, 2015).

**Table 1:** **List of material abbreviations**.

Biomaterial	Abbreviation
Hydroxyethyl methacrylate hyaluronic acid	HEMA-HA
Poly(lacto-co-glycolic acid)	PLGA
QHREDGS/collagen + chitosan	QHG213H
Polyethylene glycol	PEG
poly δ-valeracetone	PVL
RADA16-II + jagged1	RJ
Polypyrrole	PPy
Small intestinal submucosal ECM	SIS-ECM
Poly(ε-caprolacetone)	PCL
Poly(ε-caprolacetone)-2-HEMA and poly(*N*-isopropylacrylamide)	Dex-PCL-HEMA/PNIPAAm
Tetronic fibrinogen	TF
PEG fibrinogen	PF
Ascorbic acid	AA
Carboxylated PCL	cPCL
Polydioxanone	PDO
Poly-lactic acid	PLC
Poly(l-lactide-co-ε-caprolacetone)	P(LLA-CL)
Poly(*N*-isopropylacrilamide-co-propylacrylic acid-co-propyl acrylate)	p(NIPAAm-co-PAA-co-BA)

**Figure 2:**
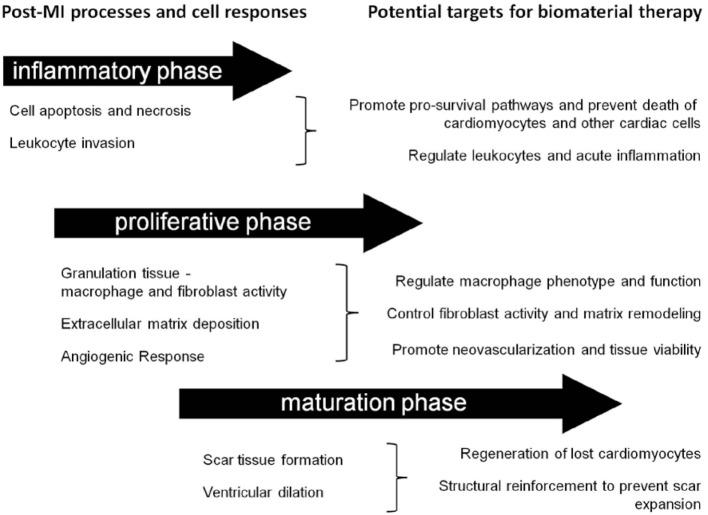
**Timing considerations for the application of biomaterial therapies**. This schematic summarizes the timeline of post-MI processes and identifies some associated potential cellular targets for biomaterial therapy.

Post-MI, the composition of the cardiac ECM undergoes considerable change as it is remodeled through the inflammatory, proliferative, and maturation phases of ischemic injury (Li et al., 2014). Since a cell’s function is controlled, in part, by interactions with the ECM environment, the cardiac ECM changes that occur post-MI can significantly affect the reparative response of the cells in the heart (Dobaczewski et al., 2011; Bayomy et al., 2012; Fan et al., 2014) (Table 2). Given the wide variety and tunability of available biomaterials, the opportunity exists to design biomaterials that can specifically target and enhance the repair function of different cell types in the MI heart (Figure 2). The goal of this review is to outline the role of four main cell types in the infarcted heart (macrophages, fibroblasts, endothelial cells, and cardiomyocytes) and provide some examples of how biomaterials have been used to alter the function of these cells to promote cardiac repair post-MI.

**Table 2:** **Cell responses and functional changes post-MI**.

	Cardiomyocytes	Macrophages	Fibroblasts	Endothelial Cells
Cellular dysfunction	– Cell death – Minimal regeneration	– M1 invasion – Inflammation	– Myofibroblast phenotype – Extended activity	– Apoptosis and necrosis – ROS production
Adverse function	– ↓Contractility – Ventricular thinning	– Excessive MMP activity – Pro-nectrotic signaling	– ECM deposition – Cardiac hypertrophy – Stiffening	– ↓ Cardiac perfusion – Sustained hypoxia

*The response of different cell types after myocardial infarction and the resultant negative outcome of these responses that may contribute to heart failure development*.

## Cardiomyocytes

### Cardiomyocytes in the Healthy Myocardium

From before birth until death, the heart is continuously working to contract and perfuse the body with oxygenated blood. To support the very high metabolic activity of the heart, the myocardium is densely vascularized to ensure adequate oxygen metabolism in order to avoid metabolic stress and the formation of reactive oxygen species (ROS). Electrical communication between cardiomyocytes occurs *via* gap junctions and is critical for regulating synchronous muscle contractions and pumping function. The myocardium also relies on the ECM for mechanical support. During diastole, collagen in the ECM passively provides stiffness to prevent temporal dilation while during systole it is able to transduce force across the myocardium (Leonard et al., 2012; Winslow et al., 2015). In addition, the interaction of cardiomyocytes with the ECM promotes survival and function (Kresh and Chopra, 2011; Okada et al., 2013).

### Function of Cardiomyocytes Post-MI

Due to the high metabolic requirements of cardiomyocytes, oxygen is one of the most important factors in heart function. During infarction, the oxygen supply is blocked or reduced to a point where the oxygen demand exceeds the supply causing excessive amounts of cardiomyocyte death. This cell death leads to a thinning of the ventricular wall in the affected area making it susceptible to rupture. In order to prevent ventricular rupture, the dead muscle is replaced with a rigid fibrous scar that does little more than provide mechanical support. As this scar does not provide much utility in the form of contractility, cardiac function continues to deteriorate over time. Although cardiomyocyte turnover in the adult myocardium has been shown to occur, these cells lack the ability to regenerate a sufficient amount of new muscle to heal the infarcted heart (Zhang et al., 2015; Awada et al., 2016). Therefore, there is an opportunity for the use of biomaterials to help boost the regenerative capacity of cardiomyocytes through the modulation of proliferation, remodeling, and survival.

### Biomaterials that Alter Post-MI Remodeling and Cardiomyocyte Properties

This section will highlight some studies that have reported on biomaterial treatments (± growth factors) with positive effects on infarct evolution and cardiomyocyte function.

#### Ligand and Cytokine-Loaded Biomaterials for Favorable Remodeling and Cardiomyocyte Function

Biomaterials can be supplemented with growth factors with the aim of preventing adverse ventricular remodeling and promoting cardiomyocyte survival and function. There are numerous examples of this strategy, of which some will be highlighted in the following sections. In one study, Cohen et al. (2014) encapsulated neuregulin-1β (NRG), a member of the epidermal growth factor family, into a hydroxyethyl methacrylate hyaluronic acid (HEMA-HA) biomaterial. Sustained release of NRG from the biomaterial was maintained for 14 days *in vitro* as the biomaterial degraded. *In vivo*, MI hearts treated with NRG/HEMA-HA showed increased NRG content within cardiomyocytes and reduced caspase-3 expression compared to hearts treated with NRG alone, suggesting that the sustained release of NRG can improve cellular uptake and confer a cardioprotective effect. After 2 weeks, animals treated with NRG/HEMA-HA had significantly improved morphology (LV area) and function (LVEF) compared to the other treatment groups. Similar work from this group examined the effect of their HEMA-HA biomaterial conjugated with engineered stromal cell-derived factor 1-α (ESA) (Macarthur et al., 2013). The issue of the chemokine’s short half-life was addressed by the encapsulation process – ESA could be released and maintain its bioactivity for greater than 28 days when loaded into the HEMA-HA hydrogel. MI hearts treated with the ESA/HEMA-HA hydrogel had reduced scar size and LV dilation, and increased LVEF compared to the controls. It has been shown that an imbalance between proteolytic matrix metalloproteinases (MMPs) and tissue inhibitors of MMPs (TIMPs) contributes to adverse cardiac remodeling post-MI (Kassiri et al., 2009). As a strategy to correct this issue, recombinant TIMP-3 (rTIMP-3) was locally administered into infarcted porcine hearts using a hyaluronic acid hydrogel. There was a concentration-dependent inhibition of MMPs followed by a decrease in left ventricular end diastolic dimension (LVEDd) and an increase in LVEF compared to hydrogel-only and saline treated controls (Eckhouse et al., 2014). After 2 weeks, infarct expansion was decreased, and interstitial MMP levels were reduced in rTIMP + hydrogel treated animals compared to all controls. This demonstrates that regulation of MMP/TIMP-mediated remodeling can be a target for preserving cardiac morphology and function post-MI.

Several studies have investigated hydrogels for the release of growth factors that can improve cardiomyocyte survival. For example, an injectable poly (lacto-co-glycolic acid) (PLGA) nanoparticle was used to bind and deliver insulin-like growth factor-1 (IGF-1), a cytokine that can promote cardiomyocyte proliferation and survival (Chang et al., 2013). IGF-1 loaded PLGA nanoparticles were injected immediately post-MI resulting in increased phosphorylation of the cardioprotective protein Akt and a reduction in the number of TUNEL^+^ apoptotic cardiomyocytes at 24-h post-injection, compared to injection of IGF-1 alone. After 3 weeks, hearts which received injection of IGF-1 nanoparticles had reduced scar size and superior LVEF compared to controls. It appears that the biomaterial treatment in conjunction with sustained IGF-1 release provided early cardioprotective effects leading to reduced remodeling and enhanced function of the infarcted heart. Another study utilized a biomaterial derived from porcine ECM in combination with an engineered hepatocyte growth factor (HGF) fragment (Sonnenberg et al., 2015). The ECM + HGF hydrogel was able to protect cardiomyocytes from serum starvation and reduce fibrotic markers in explanted cardiac cell cultures. *In vivo*, treatment with ECM + HGF hydrogel attenuated ventricular remodeling and improved function of the MI heart compared to all control groups. Ruvinov et al. (2011) designed a dual growth factor delivery alginate gel that could sequentially release IGF-1 followed by HGF upon injection. In a rat model for acute MI, treatment with the gel containing both growth factors led to less cell apoptosis, reduced scar size, and less adverse remodeling compared to those that received growth factors in saline, the biomaterial without growth factors or the saline control. Notably, Ki-67 staining suggested that more cardiomyocytes re-entered the cell cycle and proliferated in the myocardium of rats treated with the IGF-1/HGF gel compared to the other treatment groups. In another dual delivery approach for treating MI, IGF-1 and 6-Bromoinirubin-3-oxime (BIO) were packaged into gelatin nanoparticles and complexed to an alginate hydrogel (Fang et al., 2015). This was successful at prolonging the stability of the nanoparticles, as their presence was still observed by immunofluorescence at 45 days post-injection compared to nanoparticles alone, which were not detectable beyond 10 days. The injection of IGF-1 in biomaterial, BIO in biomaterial, or IGF-1 + BIO in biomaterial all lead to an increase in the function of the MI rat heart. Hematoxylin and eosin, and cardiac troponin-T staining revealed that a greater number of cardiomyocytes were present in the infarct area of animals treated with the IGF-1 + BIO biomaterial compared to the other treatment groups. Staining for proliferating cell nuclear antigen (PCNA) revealed that the combined IGF-1 + BIO therapy also lead to more cardiomyocyte proliferation. Another group co-delivered fibroblast growth factor-1 (FGF-1) and NRG to treat the MI heart by loading them into poly (lacto-co-glycolic acid) microparticles, and observed that this treatment reduced infarct size and increased scar thickness leading to superior mechanical properties of the recovering heart (Formiga et al., 2014). Furthermore, decreased fibrosis and apoptosis of cardiomyocytes was observed, and there was a trend for an increase in the number of Ki67^+^ (proliferating) cardiomyocytes with FGF-1/NRG microparticle treatment compared to unloaded microparticles.

The incorporation of specific ligands that are involved in regulating pro-survival pathways is another attractive target for enhancing the ability of a biomaterial to improve cardiomyocyte viability and function post-MI. In one study, the angiopoetin-1-derived peptide QHREDGS, capable of binding integrins, was added to a hydrogel composed of collagen (type of collagen not specified) and the polysaccharide chitosan (termed the QHG213H gel) (Reis et al., 2015). Hearts treated with QHG213H had reduced scar size and improved function compared to the control gel (no QHREDGS) and non-injected animals, which persisted to the 6-week time point. In terms of cell death and cardiomyocyte viability, QHG213H treatment resulted in fewer TUNEL^+^ apoptotic cells and significantly more cardiac troponin-T^+^ cardiomyocytes compared to controls; there was also an increase in the expression of anti-apoptotic markers and decreased pro-apoptotic gene expression. These data suggest that QHG213H treatment can preserve cardiomyocytes post-MI leading to cardiac functional and morphological improvements. Collectively, the studies discussed above demonstrate several growth factor and ligand biomaterial strategies that can improve post-MI repair through enhanced cardiomyocyte survival and/or proliferation.

Another target for biomaterial therapy in the heart is the resident population of stem/progenitor cells, which have the capacity to differentiate into new cardiac cells, including cardiomyocytes (Mayfield et al., 2014). In a study by Boopathy et al. (2014), the responsiveness of cardiac progenitor cells (CPCs) to Notch1 signaling was exploited in the design of a biomaterial therapy for treating the infarcted heart. In order to stimulate Notch1 activation in CPCs, jagged-1 (a Notch1 ligand) was incorporated at varying concentrations to the self-assembling peptide RADA16-II [1RJ or 2 RJ (concentration dependent)], which forms nanofibers at physiological temperatures. After 48 h of culture, CPCs on 1RJ hydrogels had greater expression of Hey1, a downstream target of Notch1, as well as increased expression of endothelial genes Flt1 and vWF, and smooth muscle genes Tagln and Acta2 compared to control gels. In comparison, CPCs cultured on 2RJ gels also had increased Hey1 expression, but also greater expression of cardiac genes nkx2.5, mef2c, and gata4, which was not observed with 1RJ gel cultures. Moreover, CPCs cultured on the 2RJ hydrogel for 24 h exhibited more proliferation than all controls, as determined by 5-ethynyl-2′-deoxyuridine (EdU) incorporation, indicating a role for Notch1 activation in the CPC proliferation induced with this hydrogel. *In vivo*, CPCs + hydrogels were tested in a rat MI model. Use of the 2RJ hydrogel for cell delivery resulted in greater CPC retention after 7 days, which was accompanied by improved cardiac function. There was also a trend for increased contractility in animals treated with 2RJ gels and a significant decrease in fibrosis compared to controls. In a similar approach, jagged-1 was crosslinked to fibrin nanoparticles in order to study Notch1 activation on human embryonic stem cells (hESCs) and CPCs. When cultured on the biomaterial, hESCs had increased Hey1 expression compared to culture on the nanoparticles alone, indicating a jagged-1 mediated activation of Notch1 signaling. When CPCs were plated on the jagged-1 nanoparticles, there were more cells expressing cardiac troponin-T and the cardiac transcription factor TMNT2, as well as greater formation of spontaneous beating sheets of cardiomyocytes (Tung et al., 2014). When hESCs were differentiated into cardiomyocytes and then plated on the jagged-1 biomaterial, greater cardiomyocyte proliferation was observed.

These studies demonstrate different promising growth factor- or ligand-enhanced biomaterial strategies that can (1) reduce cardiomyocyte loss, (2) stimulate cardiomyocyte proliferation within the infarct, and/or (3) promote the differentiation and/or proliferation of cardiomyocytes from stem/progenitor cell sources, ultimately leading to less adverse remodeling and improved cardiac function post-MI.

#### Biomaterial Therapy Alone for Favorable Remodeling and Cardiomyocyte Function

Biomaterials designed as meshes or injectable materials from a wide variety of natural proteins and/or synthetic polymers can also be effective treatments for MI on their own without additional growth factors or ligands. In one of the earliest of such studies, calcium cross-linked alginate was injected into the infarcted myocardium leading to a 53% increase in scar thickness compared to controls, and it improved cardiac function in a dose-dependent manner (Leor et al., 2009). In a separate study, it was also found that the alginate gel increased scar thickness and cardiac function (fractional area change, LVEF) more so than neonatal cardiomyocyte transplantation (Landa et al., 2008). More recently, alginate was crosslinked with chitosan and injected into a rat model of MI (Deng et al., 2015). The myocardium of rats treated with the chitosan–alginate gel had fewer apoptotic nuclei and less cell death compared to chitosan- or alginate-only treated animals. Notably, greater recruitment of endogenous ckit^+^ CPCs and more Ki67^+^ proliferating cardiomyocytes were observed with the chitosan–alginate treatment compared to controls, suggesting that more regeneration was stimulated with the chitosan–alginate gel. In addition to increased proliferation and recruitment, chitosan–alginate-treated animals had less adverse ventricular remodeling and superior cardiac function compared to controls. Chitosan has also demonstrated positive results when coupled with the conductive polymer polypyrrole (PPy) (Mihic et al., 2015). *In vitro*, it was shown that culture on a PPy–chitosan hydrogel could increase the rate of Ca^2+^ signal conduction in isolated neonatal cardiomyocytes compared to chitosan alone or the uncoated tissue culture plate. *In vivo*, rat MI hearts treated with the PPy–chitosan gel had increased transverse activation velocity and more efficient contractions as determined by the reduced QRS interval compared to controls. PPy–chitosan-treated animals also had reduced scar size and improved cardiac function compared to those treated with saline or chitosan alone. These results suggest that a material that enhances biological conduction can promote greater functional recovery post-MI than a non-conductive material.

**Figure 3:**
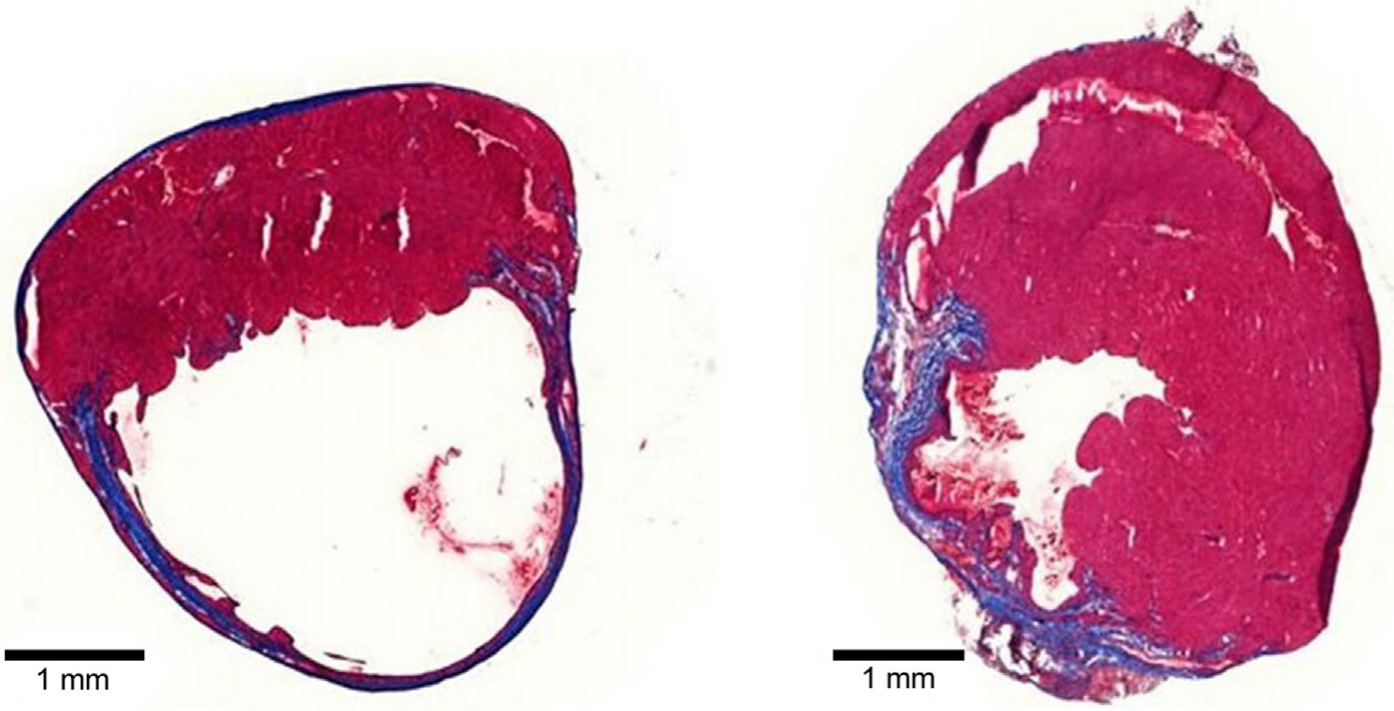
**Tissue sections of mouse MI hearts injected with PBS (left) or type 1 collagen biomaterial (right)**. Note the dilation and thinning of the collagen scar (blue tissue) in the PBS-treated heart, compared to the preservation of wall thickness and viable muscle (red) in the collagen biomaterial-treated heart.

Collagen, the most abundant ECM protein in the heart, is widely used in cardiac tissue engineering applications. Type 1 collagen-based biomaterials delivered as patches or injectable hydrogels have demonstrated the ability to reduce scar size and adverse remodeling (Figure 3) and to improve function of the MI heart (Serpooshan et al., 2013; Ahmadi et al., 2014a; Xu et al., 2014; Blackburn et al., 2015). In one study, small intestinal submucosal ECM (SIS-ECM), which is composed primarily of various collagens (type 1 most abundantly), was injected with and without circulating angiogenic cells (CACs) into a mouse model of MI (Toeg et al., 2013). Treatment with SIS-ECM alone and with SIS-ECM + CACs both lead to greater LV posterior wall thickness and reduced infarct size compared to PBS injected control animals. Newly formed cardiomyocytes and/or cardiac progenitor cells were observed in animals treated with SIS-ECM both with and without CACs. In other work, cardiac ECM, derived from the decellularization of porcine myocardium, has been shown to attenuate adverse remodeling and the deterioration of adverse cardiac function of the MI heart in both rats and pigs (Singelyn et al., 2012; Seif-Naraghi et al., 2013). In pigs, there was visible muscle retention in the endocardium of cardiac ECM-treated animals with neovascularization observed below these beds of cardiomyocytes (Seif-Naraghi et al., 2013).

Hyaluronic acid (HA)-based biomaterials have also shown promise in preserving ventricular morphology and cardiac function post-MI. For example, MI pig hearts treated with a degradable HA hydrogel (delivered 30 min after induction of MI) had decreased LV end systolic and diastolic volumes at 1-week post-treatment compared to saline controls, indicating initial preservation of function (Dorsey et al., 2015). Furthermore, there was a trend of increased function (LVEF) in HA-treated animals at all time points, which reached significance at 8 weeks post-MI. This increased function may be due, in part, to the preservation of ventricular thickness and decreased LV volumes conferred by the biomaterial at 4, 8, and 12 weeks post-MI compared to saline. The same group has also reported on the use of a HEMA-HA hydrogel containing PLGA microspheres as a bulking agent for treating the MI heart. It was found that injection of a fast-degrading HEMA-HA hydrogel containing microspheres led to increased thickness of the apical infarct area compared to HEMA-HA alone and infarct control (Tous et al., 2012). This thickening was accompanied by greater cell infiltration surrounding the PLGA microspheres and an increase in blood vessel density.

Biomaterials for treating MI have also been developed from a variety of synthetic polymers. For example, poly(ε-caprolacetone)-2-HEMA and poly(*N*-isopropylacrylamide) (PNIPAAm) were added to a biodegradable dextran chain to yield a Dex-PCL-HEMA/PNIPAAm hydrogel (Wang et al., 2009). Upon injection to the MI heart, this material formed a gel rapidly and reduced scar size while increasing ventricular wall thickness and preventing LV dilation compared to PBS injected controls. Hydrogels based on PEG, a commonly used synthetic polymer, have also been shown to have therapeutic potential for the treatment of MI. One study evaluated the effect of timing of delivery on its therapeutic efficacy (Kadner et al., 2012). It was shown that injection of the hydrogel at 1-week post-MI led to greater ventricular wall thickness compared to animals that were treated with the PEG hydrogel immediately after induction of MI (day 0). This observation was attributed to more rapid degradation of the material in the acute inflammatory environment when injected at the earlier time-point thus reducing its effectiveness as a bulking agent.

Altering the chemical composition of a biomaterial and physical characteristics such as stiffness can lead to changes in outcomes. This is demonstrated by a study in which hydrogels composed of PEG-fibrinogen (PF) were chemically modified to yield materials of different stiffness (Plotkin et al., 2014). Injection of the biomaterial to the MI heart led to an increase in LVEF compared to untreated controls with trends indicating that stiffer PF hydrogels conferred superior cardiac function. The stiffer PF was also better able to attenuate scar expansion and limit ventricular remodeling compared to the weaker PF. It should be noted that a material that is too stiff may limit the differentiation of mature cardiomyocytes or promote osteogenic differentiation (Engler et al., 2006; Forte et al., 2012). Therefore, physical properties of biomaterials can play a role in treatment outcome, and this may be dependent on the type of material, as well as the manner of its delivery.

Increasingly, researchers are investigating the use of biomaterials to assist in the differentiation and therapeutic application of cardiomyocytes derived from embryonic stem cells (ESC), induced pluripotent stem cells (iPSC), and cardiac stem cells (CSC). The issue of maturing the stem cell-derived cardiomyocyte is important and the use of biomaterials presents an attractive approach to address this. For example, in one study, a 3D type 1 collagen scaffold was developed for the co-culture of bone marrow stromal cells (BMSCs) and embryonic cardiac myocytes (Valarmathi et al., 2010). This culture system was able to yield maturing cardiomyocytes with emerging Z lines and sarcomeric fibers. An innovative engineering technique combined 3D cell culture with electrical stimulation to create biowires (Nunes et al., 2013). In this method, human iPSC were differentiated into naive cardiomyocytes and seeded on the biowire. Cells were either not stimulated or stimulated with electricity at high or low frequencies. The electrical stimulation resulted in cells adopting a rod-like phenotype (as opposed to round), indicative of cardiomyocyte maturation. Along with an increase in cardiomyocyte hypertrophy, high stimulation resulted in cells with organized sarcomeric banding and aligned Z discs. There was also a decrease in the expression of fetal genes and greater electrical coupling between cells, further confirming maturation. Another group created biomaterials using varying amounts of PEG, PCL, and carboxylated PCL (cPCL) in order to optimize a substrate for the maturation of iPSC-derived cardiomyocytes. Of the four materials tested, plating iPSC-derived cardiomyocytes on a mixture containing 4%PEG-96%PCL resulted in the greatest mitochondrial activity and strongest contractile forces generated (Chun et al., 2015). This was accompanied by an increase in the expression of cardiac genes including myosin light chain-2v and intermediate filaments known to mediate interactions with integrins. Most importantly, cardiomyocytes cultured on 4%PEG-96%PCL transitioned from expressing the fetal slow skeletal TnI (ssTnI) gene to expressing post-natal cardiac troponin-T, a hallmark of cardiomyocyte development and maturation. Depending on the particular strategy, the maturation of cardiomyocytes derived from ESC or iPSC may be a necessity before these cells can be viably transplanted.

Another application of biomaterials is in the generation and/or expansion of CSCs for therapy. For example, one group has reported on a 3D gel composed of fibrinogen and thrombin that was used to encapsulate and culture atrial cardiac tissue fragments *in vitro* (Kim et al., 2015). The biomaterial provided an ideal environment for the generation of CSCs as stromal cells appeared from the fragments after only 1 day in culture. The CSCs were viable and proliferating as determined by the incorporation of BrdU. It was also determined that the biomaterial enhanced cardiac integrin β1 signaling in CSCs compared to those cultured without the biomaterial. A system such as this may be capable of providing CSCs for further research and clinical use.

The volatile post-MI environment results in apoptosis and necrosis, the massive loss of cardiomyocytes, and adverse ventricular remodeling leading to cardiac dysfunction. There are numerous different strategies being investigated that make use of biomaterials to stimulate cardiomyocyte differentiation, proliferation and survival, providing hope that regeneration of cardiac muscle for treating MI may be possible.

## Macrophages

### Role of Native Macrophages

Macrophages are of the most abundant immune cells in the body. All macrophages, regardless of their resident location, participate in the detection of pathogens and damaged tissue, as well as the clearance of cellular debris (Pinto et al., 2014b). This occurs through macropinocytosis, a process involving a variety of pattern recognition receptors, toll-like receptor mediated identification of antigens, and the secretion of chemical factors (Lim and Gleeson, 2011). It is important to note that macrophages can be classified into two broad categories: M1 and M2 phenotype macrophages. Although this is a simplification (Frangogiannis, 2012b), there are subsets within each category (Gordon, 2003; Mantovani et al., 2004). M1 macrophages are considered to be pro-inflammatory and mediate the initial stages of inflammation after injury/insult; while M2 macrophages have an opposing function, as they are anti-inflammatory, and promote wound healing and regenerative processes such as angiogenesis to help return the tissue to its natural state (Gordon, 2003; Mantovani et al., 2004; Nahrendorf et al., 2007, 2010; Frantz and Nahrendorf, 2014; Zhang and Wang, 2014). The heart has its own resident macrophages known as cardiac tissue macrophages (cTMs). The cTMs differ from many other macrophages as they display an alternatively active, M2-like phenotype (Pinto et al., 2012). cTMs interact with other cardiac cell types and play an important role in cardiac homeostasis. It has been shown that cTMs are involved in capillarization of the myocardium, which may be achieved through neuropilin1 (NRP1) signaling and direct interaction with endothelial tip cells to promote anastomosis (Fantin et al., 2010). cMTs also play a role in controlling age-dependent fibrosis, particularly at the epicardial level (Biernacka and Frangogiannis, 2011; Pinto et al., 2014a). Maintenance of the epicardium is critical, as it contains multi-potent progenitor cells that have the ability to differentiate into endothelial cells, fibroblasts, and smooth muscle cells in the event of cardiac injury (Smart et al., 2007; Chong et al., 2011).

Evidence continues to accumulate demonstrating the importance of resident macrophages in maintaining the optimal cardiac environment. Understanding how macrophage dysfunction can lead to a variety of pathophysiological conditions is likely to improve therapies for treating the diseased heart.

### Macrophages during Myocardial Infarction

Macrophages play a vital role in the healing of cardiac tissue after infarction. It is important to remember that macrophages are a plastic cell with two main phenotypes (M1 and M2) that are derived from the differentiation of monocytes (Gordon, 2003; Mantovani et al., 2004; Zhang and Wang, 2014). The inflammatory response after MI is initiated by the release of cytokines from injured/dying cardiomyocytes, which stimulates the recruitment of neutrophils and monocytes (Sutton and Sharpe, 2015). Neutrophils and monocytes are among the first cell populations recruited to the infarct area post-MI, arriving within an hour and persisting in the myocardium for several days (Jung et al., 2013). The sustained inflammatory environment in acute MI causes the release of macrophage colony-stimulating factor (M-CSF) leading to the phenotypic transition of monocyte to macrophage (Frangogiannis et al., 2003). The polarization state of the macrophage (M1 vs. M2) depends on additional stimuli received from the cell’s environment and determines the mode of action of that particular cell (Mantovani et al., 2004). M1 macrophages are known to be pro-inflammatory and are found in cardiac tissue soon after MI. This pro-inflammatory phase is necessary to promote the removal of debris and to initiate healing from other immune cells. M2 macrophages, on the other hand, oppose the action of the M1 as they are anti-inflammatory and pro-wound healing (Nahrendorf et al., 2007, 2010; Zhang and Wang, 2014). M2 macrophages are found in significant numbers later in the infarct healing process, at approximately 7 days post-MI. M2 macrophages are associated with infarct healing, and a shift toward an increased M2:M1 ratio is beneficial for the resolution of the inflammatory state and enhancement of angiogenesis and decreased scar size (Harel-Adar et al., 2011). There are several sources for the recruited macrophages. As discussed previously, there is a resident population of macrophages within the heart tissue, but during MI, this population can become quickly exhausted. Cytokine signaling from the injured myocardium also mobilizes additional monocytes/macrophages from two other primary resources. In the bone marrow, hematopoietic stem cells are signaled through specific adhesion molecules to initiate production of mononuclear cell types needed for repair (Ehninger and Trumpp, 2011; Lo Celso and Scadden, 2011), and monocytes are stimulated to be released and home to the infarct area where they differentiate into macrophages upon arrival (Dewald, 2005). There also exists a reservoir of monocytes within the spleen, which are mobilized post-MI and contribute significantly to the macrophage population found during the MI healing process (Swirski et al., 2009). Biomaterial therapy may be an attractive strategy to regulate macrophage function in the infarcted myocardium for promoting improved repair/regeneration.

### Effect of Biomaterials on Macrophages

Being immune cells, macrophages are very sensitive to their environment. Therefore, biomaterials have the potential to vastly alter macrophage function and remodeling in the post-MI heart. Biomaterials can be designed with different compositions and properties in order to regulate macrophage polarization, proliferation, and remodeling, and several examples of this are discussed in the following section.

Several studies have investigated the effect that biomaterial composition can have on macrophage polarization. In one study, monocytes isolated from healthy human donors were cultured on several different biomaterials for 3 days prior to RNA isolation and qPCR for markers of polarization. Cells cultured on either a Parietex^™^ composite or polyethylene terephthalate yielded a high M1:M2 index indicating greater M1 polarization, whereas the culture of monocytes on polypropylene had the opposite effect: greater M2 polarization (Grotenhuis et al., 2013). This shows that macrophage polarization can be differentially regulated depending on the biomaterial administered. In another study, an *in vivo* comparison was made between a natural scaffold derived from porcine small intestinal submucosa (SIS), and a crosslinked version of SIS, known as a CuffPatch (CDI-SIS) (Badylak et al., 2008). In the SIS material, there was a strong mononuclear cell response at 1, 2, and 4 weeks after graft implantation, which was determined to be predominantly CD163^+^ M2 macrophages. Throughout the observation period, there continued to be a M2 response, and at the end of 16 weeks, the surgical area was characterized by organized skeletal muscle and collagenous connective tissue. Conversely, the crosslinked CDI-SIS patch elicited the recruitment of an equal number of M1 and M2 macrophages at 1 and 2 weeks, with the M1 phenotype predominating in later weeks, and remaining at the border of the patch rather than infiltrating into the interior. This demonstrates the potential sensitivity of macrophages to a biomaterial, as a single change to SIS resulted in drastically different cell responses. Other work has aimed to identify polarization, as well as subsequent localization, of different cell colonies in response to a variety of different biomaterial patches. In the biomaterials that predominantly stimulated M1 polarization, clusters of M1 macrophages were observed around the border of the material, while the few M2 macrophages that were present had infiltrated the material’s center and contributed to constructive remodeling (Brown et al., 2012). Of the biomaterials tested, two were successful at polarizing the majority of the cells to an M2 phenotype: the Surgisis^®^ eight-layer porcine SIS mesh and the MatriStem^®^ four-layer porcine urinary bladder scaffold. Implantation with either of these two biomaterials led to dense M2 cell infiltration in the center of the scaffolds with more organized tissue formation.

Studies have also focused on how fiber diameter and pore size in a biomaterial can alter macrophage polarization and function. For example, undifferentiated macrophages (M0) were cultured *in vitro* on PDO scaffold materials, generated by electrospinning PDO at increasing concentrations to yield increasingly thicker fibers with larger pores. Notably, greater expression of the M2 marker arginase, and reduced expression of the M1 marker iNOS, was observed on scaffolds with larger fibers and pore sizes, suggesting that larger fiber and pore size promote the differentiation of M2 macrophages (Garg et al., 2013). In terms of functionality, the M2-differentiated macrophages were more potent in promoting capillary-like formation in an *in vitro* angiogenesis assay compared to M1-differentiated macrophages. Similar findings were reported by a group using electrospun poly(ε-caprolacetone) (PCL) vascular grafts: *in vitro*, macrophages cultured on PCL with large pores underwent significantly greater M2 polarization compared to those cultured on small pore grafts, which yielded more M1 phenotype cells (Wang et al., 2014). *In vivo*, PCL grafts were transplanted into the abdominal aorta, and the macroporous grafts promoted enhanced M2 macrophage infiltration subsequently leading to further cellular infiltration and vascularization. After 100 days, there was functional smooth muscle receptive to hormonal control and complete endothelium coverage. Although neither of the above mentioned studies were applied in a cardiac setting, both provide insights into engineering techniques that could be applied to regulate macrophage phenotype and infiltration in an MI model.

Research groups have also characterized the response of macrophages on cellular vs. acellular grafts. One study using a rat abdominal defect model compared the effects of an autologous cellular and acellular patch or a cellular and acellular patch derived from the bladder of a xenogeneic pig (Brown et al., 2009). In both the autologous and xenogeneic cellular grafts, it was found that M1 polarization predominated throughout the 28-day sampling period as determined by immunohistochemical findings. Conversely, both the types of acellular patches promoted predominately M2 polarization. The increased M2 polarization was associated with more constructive remodeling of the injured area while the cellular grafts with M1 dominance showed deposition of dense connective tissue and scar formation.

It is evident that macrophages are highly responsive to their interactions with biomaterials. Macrophage phenotype and function play a significant role in the remodeling of the post-MI heart; therefore, how a biomaterial affects macrophages may ultimately decide the overall success of a biomaterial therapy. While most studies focus primarily on the polarization of macrophage phenotype in response to the biomaterial, it may be equally important to understand the remodeling effects that take place after the macrophage infiltration. It appears that the M2 macrophage phenotype can confer beneficial constructive remodeling in damaged tissues. Research has revealed much about macrophage/biomaterial interactions; however, it will be imperative to test biomaterial effects on macrophage polarization and function in relevant models of MI, as other models do not fully recapitulate the post-MI environment, which may affect both the material and the macrophages.

## Fibroblasts

### Function in a Healthy Heart

In a healthy heart, fibroblasts play a relatively minor role compared to other cardiac cells like cardiomyocytes. They function in the background as key regulators of ECM and cardiac tissue homeostasis through autocrine and paracrine signaling. Cardiac fibroblasts both produce ECM proteins and secrete a variety of factors that degrade the ECM [e.g., matrix metalloproteinases (MMPs)], making them responsible for the overall maintenance of the ECM in cardiac tissue (van Nieuwenhoven and Turner, 2013). In addition to the production of MMPs, cardiac fibroblasts secrete the tissue inhibitors of MMPs (TIMPs), which allow them to tightly regulate ECM turnover (Brown et al., 2007; Turner et al., 2010). Cardiac fibroblasts also produce a variety of matricellular proteins such as connective tissue growth factors (e.g., CTGF/CCN2), tenascins, and thrombospondins (Frangogiannis, 2012a) that do not provide structural support, but have a role in controlling cell–matrix interactions and cellular function (Bornstein and Sage, 2002). In response to stress, such as MI, fibroblasts are activated and play a key role in the resulting cardiac remodeling process.

### Dysfunction during Myocardial Infarction

The cardiac fibroblast is a plastic cell with at least two distinct phenotypes. During MI, environmental cues of stress and tension are thought to cause the phenotypic change of the fibroblast to that of a myofibroblast, which is more responsive and better able to infiltrate the infarct zone (van den Borne et al., 2010; van Nieuwenhoven and Turner, 2013). Myofibroblasts are characterized by the expression of contractile proteins such as α-smooth muscle actin (α-SMA), an increased expression of focal adhesion proteins (e.g., cadherin), and a change in the production of ECM proteins, MMPs and TIMPs (Tomasek et al., 2002; van den Borne et al., 2010). Following the phase of acute inflammation, myofibroblasts migrate and proliferate within the infarct area during the proliferative phase post-MI. The role of myofibroblasts is to replace the dead or damaged cardiomyocytes with a deposition of ECM proteins that creates the infarct scar. Initially type III collagen is deposited, which is followed by greater production of type I collagen (Cleutjens et al., 1999). Because of the resilient nature of cardiac myofibroblasts, many remain active in the infarct area during remodeling/maturation of the scar, as well as dispersed throughout the heart actively depositing collagen (van den Borne et al., 2010). Although this process of interstitium fibrosis is required to prevent perforation of the ventricular wall, over time the increased stiffness of the scar tissue is detrimental to the heart and leads to conditions such as cardiac hypertrophy and cardiomyopathy, and the deterioration of cardiac function (Opie et al., 2006; Schocken et al., 2008). Given the key role of fibroblasts/myofibroblasts in ventricular remodeling and stiffening of the myocardium, these cells are an attractive target for regulation by biomaterial therapy.

### Biomaterials Alter Fibroblast Function Post-MI

Several studies have evaluated the ability of biomaterials to modulate fibroblast function with the hope of minimizing or preventing adverse remodeling events post-MI. For example, a 3D polyethylene glycol (PEG) *in vitro* model was tested for its capacity to control fibroblast viability, migration, and function. Entrapping the cells within the PEG framework through covalent crosslinking and gelation of the material did not appear to harm the cells as viability in the range of 90–95% was observed after this process (Raeber et al., 2007). It was found that the migration of fibroblasts within PEG was significantly reduced in the presence of MMP inhibitors, indicating that fibroblast migration was dependent on MMPs. MMP activity was deemed necessary to digest the PEG’s pores of nanoscale size that otherwise restrict the passage of cells. In terms of function, fibroblasts were observed to form interconnected 3D cellular networks within the PEG hydrogel over a period of several weeks, demonstrating their capability for long-term survival within the material. Another study aimed to measure the effect of varying hydroxyapatite particle size on fibroblast proliferation. At all time points, a significantly greater number of cells was observed on the hydroxyapatite with larger particle size (Sun et al., 1997), suggesting that greater particle size provides the cells with more space to proliferate. These studies demonstrate that physical space within a material can have an effect on cell behavior.

The composition, and more specifically the interactive sites, of a biomaterial can also regulate fibroblasts activity. For example, in 3D PEG networks, the incorporation of the cell-adhesion motif RGD led to significantly increased fibroblast proliferation compared to hydrogels without the motif. Proliferation was also determined to be associated with biomaterial stiffness, independent of RGD or MMP sensitivity of the hydrogel: as the elastic modulus (stiffness) of the material was increased, fibroblast proliferation significantly decreased over a period of 21 days (Bott et al., 2010). This observation *in vitro* suggests that increased stiffness of the collagenous scar post-MI may be a factor contributing to the minimal cellularity of the scar. It may be worth directing research toward ways of softening the scar to improve its recellularization.

In an *in vitro* model for blood vessel regeneration, a dual-layer composite biomaterial consisting of poly-ε-caprolacetone as the inner layer and poly-lactic acid as the outer layer was created by electrospinning (Vaz et al., 2005). Fibroblasts proliferated along the length of the tubular scaffold after 1 week, and after 1 month, confluence was achieved and myofibroblasts congregated more closely at the surface of the scaffold. Myofibroblasts cultured on the tubular scaffold deposited less ECM proteins and glycosaminoglycans compared to a control porcine pulmonary valve material. Although not tested in the cardiac setting, this suggests that the composite biomaterial may have the ability to limit the ECM remodeling function of myofibroblasts, which may have the potential to decrease cardiac fibrosis post-MI if applied at the opportune time (i.e., after the susceptibility to rupture has passed). Another group used electrospun silk fibroin nanofibers coated with different ECM proteins to see how fibroblasts response would be affected. Fibroblasts were seeded on tissue culture polystyrene, silk fibroin, bovine serum albumin, type 1 collagen-coated silk fibroin, fibronectin-coated silk fibroin, or laminin-coated silk fibroin. The cells that adhered to collagen- and laminin-coated silk fibroin exhibited a spreading morphology characteristic of proliferating fibroblasts more so than the cells in the other substrate groups (Min et al., 2004). It is evident from the numerous culture studies that materials can be modified to differentially regulate the function of fibroblasts.

While promising results are being generated *in vitro*, there are many factors present in the post-MI heart *in vivo* that are not reproduced in the culture models. Therefore, establishing the effects of a biomaterial on fibroblast activity and function using animal models of MI is critical. One particularly good example of this comes from a study in which alginate was modified by covalently adding RGD and YIGSR adhesion domains to provide cell adhesion sites. *In vitro*, fibroblasts displayed strong adhesion to the modified biomaterial, which was predicted to enhance the cell response *in vivo* (Tsur-Gang et al., 2009). However, the peptide-modified materials exhibited reduced therapeutic potential when applied in a rat MI model compared to the unmodified alginate. No differences were observed in the infiltration of fibroblasts or cell proliferation between the different alginate formulations after 8 weeks. It was postulated that the peptide-modified alginate may undergo additional mechanical changes upon injection *in vivo* that may affect structure and stiffness, thus altering its physical support properties as well as its influence on cell function. In another study, fibroblasts cultured on a type 1 collagen–chitosan hydrogel had less α-SMA expression and less deposition of fibrillar collagen than cells cultured on the collagen-only hydrogel (Ahmadi et al., 2014b). *In vivo*, intramyocardial injection of the chitosan–collagen material to MI mouse hearts resulted in less fibrosis, superior scar thickness, and improved cardiac function compared to hearts treated with the collagen only hydrogel. This improved therapeutic effect was associated with an altered MMP9/TIMP2 expression profile that likely contributed to the reduction in adverse remodeling.

In summary, fibroblasts play an important role in MI wound healing and therefore enhancing their function post-MI may be what is needed to significantly improve outcomes in patients with MI. Promising *in vitro* work suggests that fibroblasts are highly responsive to a variety of biomaterials and are able to adhere and proliferate on many of them. However, more *in vivo* evaluation is needed in order to assess how fibroblasts will respond to materials in the MI setting. The stressed and changing post-MI environment introduces infinitely more variables that need to be considered when selecting and tailoring biomaterials for implantation. Many technologies are available for the customization of different biomaterial properties from fiber and pore size to mechanical properties like stiffness and elasticity, with the aim of better controlling fibroblast function *in vivo*. However, a better understanding of the mechanisms involved in fibroblast-material interactions will be needed in order to effectively apply these technologies.

## Endothelial Cells

### Endothelial Cell Function

Endothelial cells line the inner lumen of all the vasculature and contribute to the regulation of blood flow. In larger blood vessels, chemical signals from the blood are detected by endothelial cells and communicated to the surrounding smooth muscle for the regulation of vasomotor tone and blood flow. For example, nitric oxide signaling from the endothelial cell stimulates smooth muscle cells leading to vasodilation and increased blood flow (Cines et al., 1998; Michiels, 2003; Deanfield et al., 2007). Endothelial cells also respond to mechanical stimuli, such as shear strain, to secrete vasoactive factors that can alter the physiological function of distant target organs or mobilize cell populations for recruitment if needed (Michiels, 2003; Chien, 2007; Deanfield et al., 2007). One of the most important functions of endothelial cells is their ability to act as a barrier to prevent the unwanted infiltration of foreign substances. The endothelial cell is recognized as a key to maintaining vascular homeostasis so that the body can function with optimal hemodynamics.

### Vascular Pathophysiology during MI

During the hypoxia that follows MI, a significant loss of endothelial cells through apoptosis and necrosis occurs, much like the other cells of the heart. The cells that do survive experience oxidative stress, which severely alters endothelial cell physiology. The presence of reactive oxygen species (ROS) increases the expression of cell adhesion molecules and the permeability of endothelial cells leading to an enhanced recruitment/invasion of leukocytes (Lum and Roebuck, 2001). The recruited immune cells, such as macrophages, amplify the pro-necrotic signaling in the local hypoxic environment resulting in more tissue death (Nahrendorf et al., 2007, 2010). The necrosis of vascular tissues means that even when blood flow is restored to the coronary system, the infarcted tissue can remain inadequately perfused, leading to further damage and infarct expansion. Therefore, therapies that limit vascular death and/or improve the regenerative capacity of the endothelium are expected to better preserve cardiac function post-MI.

### Enhancing Endothelial Cell Function with Biomaterials

Angiogenesis is a critical component of the healing process post-MI. Without adequate revascularization, hypoxic or anoxic conditions persist in the cardiac tissue leading to more cell death over time. Furthermore, the regeneration of blood vessels is paramount to the regeneration of the heart as a whole. Blood vessels are required to transport oxygen and nutrients to the cells, as well as to remove toxic waste products. Many biomaterials have shown promise as a strategy to improve the function, proliferation, and survival of endothelial cells in an attempt boost angiogenesis post-MI.

Several studies have reported on the performance of an electrospun nanofiber scaffold composed of poly(l-lactide-co-ε-caprolacetone) [P(LLA-CL)] (Mo et al., 2004; Xu et al., 2004; He et al., 2005). The biomaterial is fabricated such that the – CL unit is highly organized on each nanofiber of the scaffold. *In vitro*, smooth muscle cells and endothelial cells seeded onto the matrix adhered after 1 day and significant proliferation was observed by day 7 (Mo et al., 2004). When the alignment of the material was made to more closely resemble the native formation of typical arteries, smooth muscle cells elongated along the length of the nanofiber in spindle-like fashion after 5 h of culture, and after 3 days, they appeared bi-polar indicating a contractile phenotype (Xu et al., 2004). Although not dealing directly with endothelial cells, this study shows that the P(LLA-CL) material is capable of supporting cells needed for blood vessel growth/maturation during angiogenesis. In another study, the P(LLA-CL) was used as a nanofiber mesh, which was coated in type 1 collagen prior to seeding with human coronary artery endothelial cells (HCAECs) (He et al., 2005). HCAECs adopted a flattened, more spread out morphology on the collagen-coated network than on the uncoated network, which is indicative of an adherent phenotype necessary for vascular grafting. While *in vitro* testing yields important information about endothelial cell-biomaterial interactions, *in vivo* studies are needed to properly evaluate the potential for a biomaterial to support blood vessel regeneration. Many factors *in vivo* could affect the performance of a biomaterial, such as interactions with other cell types, pH changes, hypoxia, and the presence of degrading enzymes.

In one study, a porous bovine pericardial patch containing MSCs was able to enhance the function of endothelial cells when implanted into a right ventricular defect. Greater capillary growth was observed in the inner and outer layers of the MSC patch compared to the control patch without MSCs at 4 weeks post-implantation; and there was an intact layer of endothelial cells on the inner layer of the MSC patch, which did not occur in the control (Wei et al., 2006). Transplanted MSCs were shown to differentiate into endothelial cells and smooth muscle cells that contributed directly to new capillary formation; although capillary density was reduced compared to the healthy myocardium. Similar results were obtained with injection of a fibrin glue scaffold. The fibrin material limited adverse remodeling and increased blood vessel regeneration within the infarct zone compared to control BSA injections (Christman et al., 2004). In a study comparing fibrin, collagen type 1, and Matrigel, it was found that all three materials promoted angiogenesis and increased capillary density compared to PBS-injected control animals (Huang et al., 2005). All the materials produced positive results that were not significantly different from each other. These results suggest that endothelial cells are a relatively invasive cell that can populate the MI area if provided with a suitable substrate or environment, such as a biomaterial scaffold.

The supplementation of a biomaterial with growth factors is an approach that has been tested often for enhancing vascular regeneration. For example, in one study, a collagen hydrogel composed of type I and type 3 collagen was crosslinked and conjugated with VEGF prior to subcutaneous injection, and tested against pure collagen, crosslinked collagen, and VEGF-collagen (He et al., 2011). There was greater cell infiltration in the crosslinked VEGF-conjugated collagen compared to pure collagen at 1-week post implantation. At 2 weeks, the number of microvessels in the crosslinked VEGF conjugated collagen was significantly greater than all other groups. It was found that there was a greater amount of VEGF immobilized by crosslinking, and that angiogenesis was sustained for a longer period of time compared to the other three groups. In a similar strategy, collagen patches (Ultrafoam^™^ collagen sponge scaffolds; Davol) with VEGF covalently bound at low or high concentration were implanted into MI hearts. Both materials were able to preserve ventricular wall thickness, with the greater thickness observed in the high VEGF biomaterial (Miyagi et al., 2011). After patch engraftment to the infarcted region, bone marrow cells were injected to the biomaterial-treated heart; increased retention and proliferation of the transplanted cells, as well as greater vascular density was seen in the high VEGF biomaterial group compared to control patches and low VEGF patches. Similar to VEGF, the inclusion of other angiogenic factors, such as FGF-2, IGF-1, and HGF, in a biomaterial has also led to increased vascularization of cardiac tissue. It has been shown that the sequential release of IGF-1 followed by HGF from an affinity-binding alginate hydrogel was able to increase angiogenesis and mature blood vessel formation in the MI heart more so than single growth factor control gels or gels without growth factors, suggesting that the timing and selection of growth factors for delivery is an important consideration (Ruvinov et al., 2011). Another group developed a poly(*N*-isopropylacrilamide-co-propylacrylic acid-co-propyl acrylate) [p(NIPAAm-co-PAA-co-BA)] material that was supplemented with biotinylated FGF before being injected into infarcted hearts. A 10-fold increase in FGF retention was observed in the p(NIPAAm-co-PAA-co-BA)-FGF group compared to animals that received FGF delivered in saline, which led to ~40% greater arteriolar and capillary density (Garbern et al., 2011). After 28 days, there was a twofold increase in perfusion of the infarcted area in hearts treated with the polymer + FGF, whereas controls showed no improvement in perfusion. Overall, many studies have demonstrated that growth factor supplementation of a biomaterial is a promising therapeutic approach for the regeneration of blood vessels.

Autologous *in vitro* tissue engineering is another technique under investigation for generating vascularized tissue. With this type of biomaterial, cells are isolated from the donor and cultured *in vitro* in a tissue-engineered scaffold to generate new tissue that can later be implanted back into the host. In one example, human osteoblasts and human micro-capillary endothelial cells were seeded onto a silk fibroin scaffold *in vitro*. This resulted in the formation of a micro-capillary network intertwined with scaffold and osteoblast cells (Unger et al., 2007). To test its functionality, the biomaterial was implanted subcutaneously into immunodeficient mice. After 14 days, the scaffold was removed and it was determined that the micro-capillary network that was formed *in vitro* successfully connected with the host vasculature and became perfused. The silk fibroin scaffold also promoted the migration of host vasculature into the biomaterial (Unger et al., 2010). This demonstrates the viability of *in vitro* pre-vascularized biomaterials as a strategy to quickly restore vascular networks within damaged tissues. In a more cardiac-relevant model, a hydrogel made from decellularized porcine heart ECM was engineered to gel at physiological temperature and self-assemble into a complex structure post-injection (Singelyn et al., 2009). Injection of the cardiac mimicking biomaterial lead to significantly increased arteriole density compared to fibrin controls.

There are many promising biomaterial strategies that have demonstrated success in improving the function of endothelial cells and stimulating revascularization *in vitro* and *in vivo*. Despite this, more studies are needed that address the organization, maturation and persistence of the newly formed vascular networks in relevant models of coronary artery disease.

## Future Directions

Emerging technologies such as 3D printing and nanoparticles may provide the opportunity for new biomaterial manufacturing techniques to further advance the field of regenerative medicine. One group has demonstrated success with bioprinting a 3D alginate/gelatin hydrogel network. Cells embedded within this construct were capable of controlling the degradation of the biomaterial in the presence of sodium citrate, which in turn allowed the cells to have superior proliferation and differentiation (Wu et al., 2016). Another group created bioink derived from decellularized adipose tissue (DAT) to make 3D printed biomaterials (Pati et al., 2015). It was shown that human adipose tissue-derived mesenchymal stem cells encapsulated within the DAT printed biomaterial matured normally with high viability without the addition of supplemental adipogenic factors. Upon sub-cutaneous implantation in mice, the cell-seeded construct supported positive tissue infiltration and constructive remodeling, and was non-inflammatory. Physical properties of a material, such as substrate modulus, may be specifically tailored by using 3D printing. Guo et al. showed that a poly(ester urethane) biomaterial with a substrate modulus similar to collagen fibers was able to reduce inflammation, increase angiogenesis, and decrease fibrosis when used to treat dermal wounds. The material elicited a down-regulation of Wnt/β-catenin signaling in fibroblasts, and increased M2 polarization of macrophages, demonstrating their ability to promote a regenerative, rather than a fibrotic/scar response in host cells (Guo et al., 2015). In a cardiac model, a 3D printed gelatin/HA biomaterial loaded with human cardiac-derived progenitor cells was applied post-MI in mice leading to a reduction in adverse remodeling and preservation of cardiac function (Gaetani et al., 2015). The transplanted cells exhibited enhanced survival over a period of 4 weeks, as well as increased expression of cardiac and vascular differentiation markers.

Another increasingly studied approach for regenerative medicine involves the use of nanoparticles. For example, Baei et al. used gold nanoparticles in combination with a chitosan hydrogel to create a material with the potential to support electrical conduction in electro-active tissues. MSCs were seeded onto the gold nanoparticles-chitosan hydrogel for 14 days and it was found that the biomaterial supported normal cell metabolism, migration and proliferation as well as increased cardiomyogenic differentiation (Baei et al., 2016). In another application, decellularized vascular tissue was conjugated with gold nanoparticles and used in patch angioplasty of the carotid artery in mice. The vascular patch with gold nanoparticles showed increased endothelial regeneration and a normal healing response with excellent tissue integration (Ostdiek et al., 2016). Both 3D printing and nanoparticle technology have shown promise in the regenerative field but like other biomaterial approaches, they require more research and optimization before they can be clinically employed.

## Conclusion

It has been shown that biomaterials are capable of enhancing the repair/regenerative capacity of many different cell types (summarized in Table 3). Knowing that different cells are dominant as the infarct evolves through its inflammatory, proliferative, and maturation phases will help in designing and optimizing materials for application at different time-points post-MI. A biomaterial can influence cells either directly or indirectly through downstream signaling and knowing these signaling mechanisms will also allow for greater control of the healing process. This review highlights the abilities of biomaterials to enhance cells and repair by focusing on each cell type individually; however, materials are likely to affect more than a single cell type when administered into the MI heart so that multiple repair processes can be targeted simultaneously. It may also be that multiple biomaterial strategies will need to be applied in combination to promote appropriate repair responses from the different cell types. With improving engineering technologies, it is possible to customize biomaterials to possess specific physical and biological parameters in order to optimize host cell function. Successful biomaterial therapy will be able to improve cardiac function at a cellular level, which would improve patient outcomes post-MI.

**Table 3:** **Cell responses and associated functional benefits that have been observed with different biomaterial therapy strategies**.

	Cardiomyocytes	Macrophages	Fibroblasts	Endothelial Cells
Cellular improvements	↓Apoptosis	↑Recruitment	↑Proliferation	↑Proliferation
	↑Proliferation	↑M2 polarization	↑Migration	↑Cell survival
	↑Recruitment of cardiac repair cells		↓Myofibroblast activity	↑Vessel retention
	↑Ca^2+^ conduction			↑Cell localization
Functional benefits	↓Remodeling	↓MMP Activity	↓ Fibrosis	↑Formation of neocapillaries
	↓Scar expansion	↑Neovascularization		↓Vessel remodeling
	↑LVEF			↑Vessel regeneration and angiogenesis
	↑Ventricular wall thickness			↑Vascular density
	↑Fractional shortening			↑ Perfusion

## Author Contributions

ZL, KR, and ES were responsible for the conceptualization, writing and/or editing of the manuscript. KR and ES provided supervision.

## Conflict of Interest Statement

The authors declare that the research was conducted in the absence of any commercial or financial relationships that could be construed as a potential conflict of interest.
